# Internal limiting membrane peeling versus no peeling during primary vitrectomy for rhegmatogenous retinal detachment: A systematic review and meta-analysis

**DOI:** 10.1371/journal.pone.0201010

**Published:** 2018-07-19

**Authors:** Matteo Fallico, Andrea Russo, Antonio Longo, Alfredo Pulvirenti, Teresio Avitabile, Vincenza Bonfiglio, Niccolò Castellino, Gilda Cennamo, Michele Reibaldi

**Affiliations:** 1 Department of Ophthalmology, University of Catania, Catania, Italy; 2 Department of Biomedical and Biotechnological Sciences, Pathology and Oncology Section, University of Catania, Catania, Italy; 3 Department of Clinical and Experimental Medicine, University of Catania, Catania, Italy; 4 Eye Clinic, Department of Neurosciences, Reproductive Sciences and Dentistry, University of Naples Federico II, Naples, Italy; Universita degli Studi di Firenze, ITALY

## Abstract

**Background:**

Internal limiting membrane (ILM) peeling during primary vitrectomy for rhegmatogenous retinal detachment (RRD) prevents the formation of postoperative macular epiretinal membrane (ERM). However, studies that compared vitrectomy with and without ILM peeling for RRD, have reported controversial outcomes.

**Objective:**

To assess the efficacy of ILM peeling versus non-ILM peeling during vitrectomy for RRD by a systematic review and meta-analysis of published studies.

**Methods:**

PubMed, Medline, Web of Science, Embase databases, and the Cochrane Library were searched up to April 2018 to identify studies that compared primary vitrectomy with and without ILM peeling for RRD with at least six months follow-up. Primary outcomes were the rate of postoperative ERM formation and mean best corrected visual acuity (BCVA) change after vitrectomy. Rate of recurrence of retinal detachment (RD) was assessed as secondary outcome. Risk ratios (RRs) with 95% confidence intervals (CIs) expressed pooled results for rate of ERM formation and rate of RD recurrence in ILM peeling and non-ILM peeling groups. Pooled results for BCVA change in the two groups were expressed as Weighted Mean Difference (WMD) with 95% CIs.

**Results:**

Nine studies, one of which was a randomized controlled trial (RCT), with a total number of 404 eyes in the ILM peeling group and 365 eyes in the non-ILM peeling group, were included. The analysis from pooled data indicated a significant lower rate of postoperative ERM formation in the ILM peeling group compared to the non-ILM peeling group (9 studies, 769 eyes, RR = 0.14; CI: 0.07 to 0.28; P < 0.001). There was no statistical difference in mean BCVA change (9 studies, 769 eyes, WMD = 0.02; CI: -0.11 to 0.16; P = 0.75). Rate of recurrence of RD was lower in the ILM peeling group (6 studies, 603 eyes, RR = 0.32; CI = 0.17 to 0.61; P< 0.001).

**Conclusion:**

ILM peeling during vitrectomy for RRD prevents the formation of macular epiretinal membrane postoperatively and reduces the incidence of RD recurrence, but better visual outcome was not found compared to non-ILM peeling vitrectomy.

## Introduction

Since the introduction of small gauge vitrectomy, a broad change in the treatment of rhegmatogenous retinal detachment (RRD) has taken place, as a shift towards pars plana vitrectomy has made this the preferred procedure with respect to scleral buckle [1].

Consequently, ever-increasing attention has been paid to possible complications related to this surgery and how to limit unpleasant events. Macular epiretinal membrane (ERM) represents one of the most common visual impairing complications after vitrectomy for RRD repair, with an incidence reported between 6% and 48% [2–11] and further surgery required for ERM to be removed in one third of cases [12].

Mechanisms involved in the pathogenesis of ERM secondary to vitrectomy for RRD seem to be different in comparison with idiopathic ERM. Retinal pigment epithelial cells have been attributed a key role, migrating from retinal breaks towards the macular surface, on which they proliferate. In this context internal limiting membrane (ILM) has been assumed to act as a scaffold for the ERM to be built up [3,12].

For these reasons, peeling the ILM has been postulated to remove ERM originator cells from the macular surface, reducing the risk of postoperative ERM formation [12].

On this basis, some authors described ILM peeling during primary vitrectomy for RRD with the purpose of reducing ERM development [3–11]. These studies agree in demonstrating a significant reduction in the incidence of ERM after vitrectomy with peeling of the ILM, even though a clear discrepancy exists regarding the effect of peeling on visual acuity; in some of these studies, better visual results were reported in eyes receiving ILM peeling compared to non-ILM peeling, whereas in others no difference was found between the two procedures, and in the only randomized trial, a significantly worse functional result was described after ILM peeling [3–11].

The effect of ILM peeling in these cases remains a debatable issue and to date no systematic review with meta-analysis has been published.

Therefore, given the lack of agreement on the necessity of ILM peeling, along with limitations presented by published studies, we conducted a systematic review and meta-analysis of the literature with the aim of assessing functional and anatomical effects of ILM peeling versus non-ILM peeling during primary vitrectomy for RRD.

## Materials and methods

### Literature search methods

This analysis was conducted in accordance with the Preferred Reporting Items for Systematic Reviews and Meta-analyses (PRISMA) guidelines (S1 Table) [13] and the methods described in the Cochrane Handbook [14]. Studies comparing the outcomes of primary vitrectomy with ILM peeling versus non-ILM peeling for RRD were systematically reviewed. PubMed (from inception up to 29 April 2018), Medline (1946 to 29 April 2018), Web of Science (1985 to 29 April 2018), Embase databases (1980 to 29 April 2018), and the Cochrane Library (searched 29 April 2018) were searched. The electronic search method included the terms ‘internal limiting membrane’, ‘inner limiting membrane’, ‘ILM’, ‘vitrectomy’, ‘peeling’, ‘retinal detachment’, which were connected in various combinations by ‘or’/’and’ (detailed search strategy is shown in S2 Table). The last search was carried out on April 29, 2018. All relevant, potentially eligible studies, published in peer-reviewed journals and in English, were considered, irrespective of publication status, publication date or primary outcome. A manual search of reference lists of all included articles and consultation with experts in this field were also made. When clarifications were needed to assess the eligibility of the studies, we contacted the authors by email.

### Criteria of inclusion, exclusion and outcomes of interest

To be selected and enrolled for the analysis, studies had to meet the following inclusion criteria: (1) to compare clinical outcomes of primary vitrectomy with and without ILM peeling in adult patients affected by RRD; (2) to provide at least one of the two primary outcome measures considered by this meta-analysis; and (3) to present a 6 month or longer follow-up. The following exclusion criteria were adopted: case report design; cohorts including patients with a history of previous surgery except for cataract surgery, or suffering from other visual impairing diseases apart from RRD; cohorts including patients receiving vitrectomy for recurrence of retinal detachment, for retinal detachment secondary to macular hole, or for type of retinal detachment different from the rhegmatogenous one.

When the same authors and/or Institution published multiple studies with the same cohorts, we included either the most recent or the one with the best quality in this analysis.

The primary outcome measures, which were extracted for the analysis, were the rate of postoperative macular ERM development and mean best corrected visual acuity (BCVA) change after vitrectomy. Rate of recurrence of retinal detachment (RD) was assessed as a secondary outcome.

### Data extraction and quality assessment

Titles and abstracts of eligible studies were independently reviewed by two investigators (A.R., M.F.), performing full-text evaluation when inclusion criteria were satisfied. The same two investigators (A.R., M.F.) comprehensively analyzed and independently extracted the data from each included study. Disagreements were addressed by discussion, but when this failed, a third senior investigator (A.L.) was involved to achieve consensus. Data extracted from each selected paper included: year, location and first author of the study, study design, population characteristics including mean age, race, gender, number of patients, follow-up time, and proliferative vitreoretinopathy grade. The following data were extracted for each cohort: number of patients, macula status at time of surgery, BCVA change after surgery, rate of macular ERM development, and rate of recurrence of retinal detachment. Additionally, from the cohort of patients receiving vitrectomy with ILM peeling, information about whether a dye was used for peeling and, in these cases, which type of dye, was collected. When clarification or further information was required, the authors were contacted.

All information on papers, including outcomes, was collected for assessment of risk of bias. Quality of selected studies was assessed by two reviewers (A.L., V.B.) according to the Newcastle-Ottawa Scale [15]. Randomized controlled trials were evaluated by the same two reviewers (A.L., V.B.) using the Cochrane Collaboration Reviewers’ Handbook for Systematic Reviews [16]. Any potential publication bias was assessed by visual inspection of funnel plots along with Egger’s regression test and Harbord’s modified test.

### Statistical analysis

Meta-analysis was performed on the primary and secondary outcome measures. Summary estimates, including 95% confidence intervals (CIs), were calculated. For discontinuous outcomes, such as proportion of patients who were diagnosed with ERM secondary to vitrectomy and the proportion of patients with recurrence of retinal detachment, a Risk Ratio (RR) with 95% CIs was calculated. Unfavorable events (ERM formation and RD recurrence) were used as the risk numerator. For mean BCVA change, which was analyzed as a continuous variable, Weighted Mean Difference (WMD) with 95% CIs was reported. Heterogeneity was assessed by using the chi-square test and I^2^ statistic. An I^2^ value > 50% was considered as moderate to high heterogeneity. Random-effects models were used as the primary approach to meta-analysis, except when only two studies were included (fixed effects used). Harbord’s modified test was used to test asymmetry within dichotomous outcomes, whereas Egger’s regression within continuous outcomes [17]. A P value < 0.05 was considered significant for all analyses. Meta-analysis was conducted using Stata software, version 15.2 (StataCorp, College Station, TX).

## Results

### Study selection

**Fig 1:**
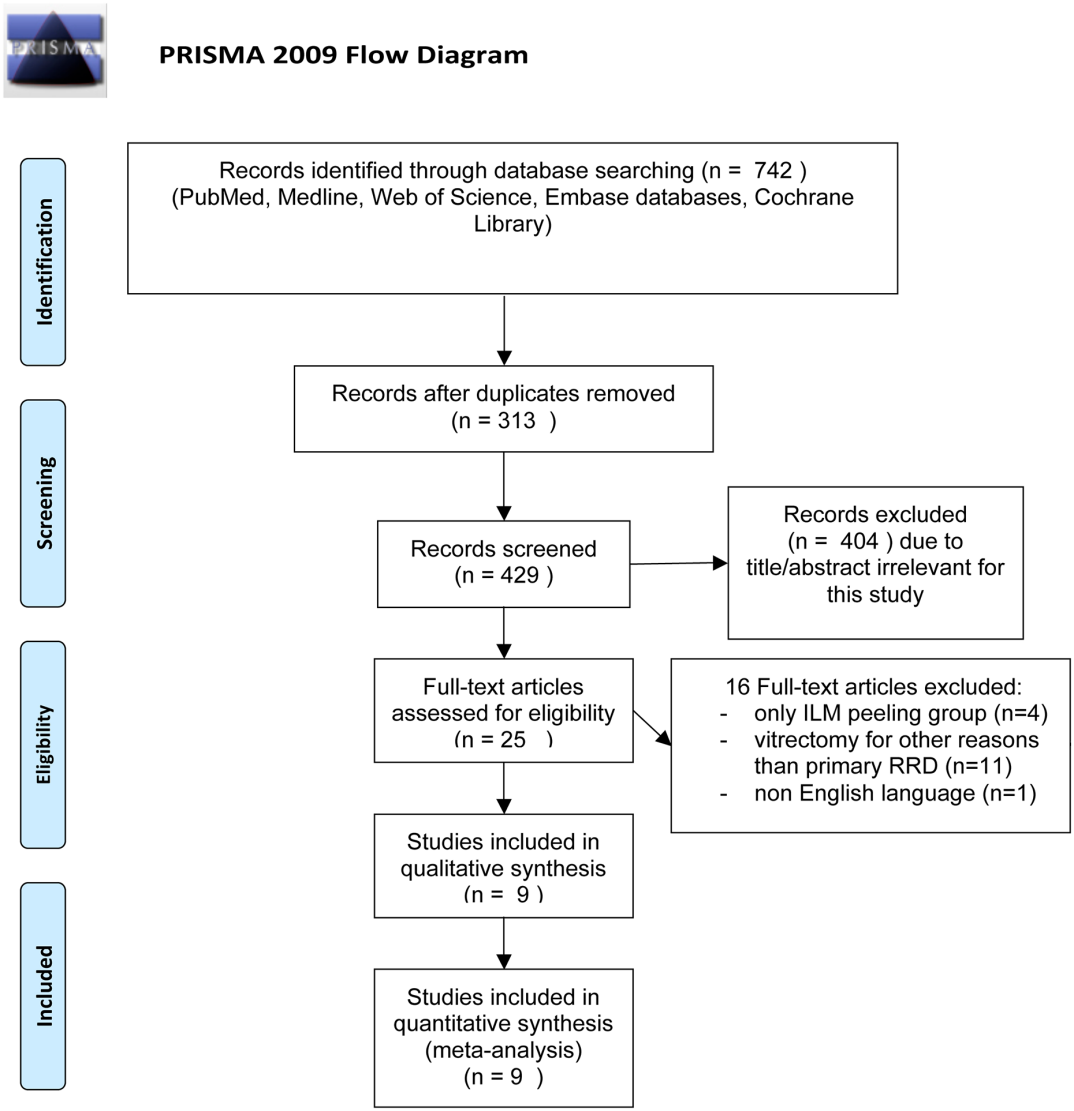
Flow diagram of the study selection process. *From*: Moher D, Liberati A, Tetzlaff J, Altman DG, The PRISMA Group (2009). *P*referred *R*eporting *I*tems for Systematic Reviews and *M*eta-*A*nalyses: The PRISMA Statement. PLoS Med 6(7): e1000097. doi: 10.1371/journal.pmed1000097 **For more information**, **visit** www.prisma-statement.org.

The overall study selection process is shown by Fig 1. A total of 742 studies were identified from the electronic database search, of which 313 were duplicates. The remaining 429 articles were screened by applying inclusion and exclusion criteria, and 25 potentially relevant studies were identified. Sixteen reports were ruled out after full-text assessment, of which 11 included eyes with a diagnosis that was different from primary RRD, one was not written in English, 4 were not comparative. A total of 9 studies were selected and included in our analysis.

### Study characteristics

Overall, this meta-analysis included 9 studies, with a total number of 404 eyes in the ILM peeling group and 365 eyes in the non-ILM peeling group. The first study was published in 2008, the last one in 2018. Eight were retrospective studies, while one was a randomized controlled trial. The minimum follow-up was 6 months, while the maximum exceeded 40 months. All studies reported the rate of macular ERM formation following vitrectomy for RRD and baseline and postoperative BCVA in the two groups. Six studies reported the rate of RD recurrence. All studies described macular status at the time of RRD diagnosis for both groups. In 7 studies dyes were used for ILM peeling: brilliant blue in 4 cohorts, trypan blue in one cohort, membrane blue in one cohort, and indocyanine green in one cohort. In one study triamcinolone acetonide assisted ILM peeling. In another one, ILM peeling was performed with and without triamcinolone acetonide assistance, but without any dye.

Not surprisingly, included studies featured different designs and clinical variables. Eight were retrospective and one was an RCT, which was conducted by Eissa et al [6] and enrolled only macula off RD. Similarly, Garweg et al [8], Foveau et al [10], Blanco-Teijeiro et al [11] and Aras et al [9] included only macula off RD. The rate of macula off RD recorded by Rao et al [7], Nam et al [3], Akiyama et al [5] and Forlini et al [4] was 74%, 44%, 42% and 68%, respectively. Eissa et al [6], Garweg et al [8] and Aras et al [9] used silicone oil as endotamponade in all cases, while Foveau et al [10], Blanco-Teijeiro et al [11], Rao et al [7] and Nam et al [3] performed vitrectomy with gas in each case. Forlini et al [4] used both tamponades. Akiyama et al [5] did not report on type of tamponade. Baseline visual acuity was also different: Nam et al [3] and Akiyama et al [5] registered a mean BCVA < 1 logMar in both the ILM peeling and non-ILM peeling group, while it was > 1 logMar in both groups of all other studies [4,6–11]. This difference in clinical aspects has to be taken into account as it might have an influence on outcomes, in particular on the functional one.

### Quality assessment and risk of bias

S3 Table displays the quality score of the 8 retrospective studies according to the Newcastle-Ottawa scale. All studies had a quality score ≥ 6, showing a low-to-moderate risk of bias. In particular, all studies were given 3 stars out of 4 for Selection category and one star out of 2 for Comparability category, since there was no bias related to representativeness of the cases, selection and definition of controls, as well as comparability of cases and controls on the basis of the design for classification of RD was adequate. Six studies scored 3 stars out of 4 for Exposure category, since exposure ascertainment was not based on a blind structured interview. Two studies obtained 2 stars out of 4 for Exposure category, as exposure ascertainment was not based on a blind structured interview and there was a different method of ascertainment for cases and controls. The Cochrane Collaboration Reviewers’ Handbook revealed an unclear risk of bias in the randomized controlled trial. No evidence of publication bias was suggested by the shape of the funnel plots (nearly symmetrical). Harbord’s modified test was not significant for rate of ERM development (P = 0.29) (S1 Fig) and rate of RD recurrence (P = 0.68) (S2 Fig), as well as Egger’s regression test for mean BCVA change (P = 0.1) (S3 Fig).

### Rate of macular epiretinal membrane development

**Fig 2:**
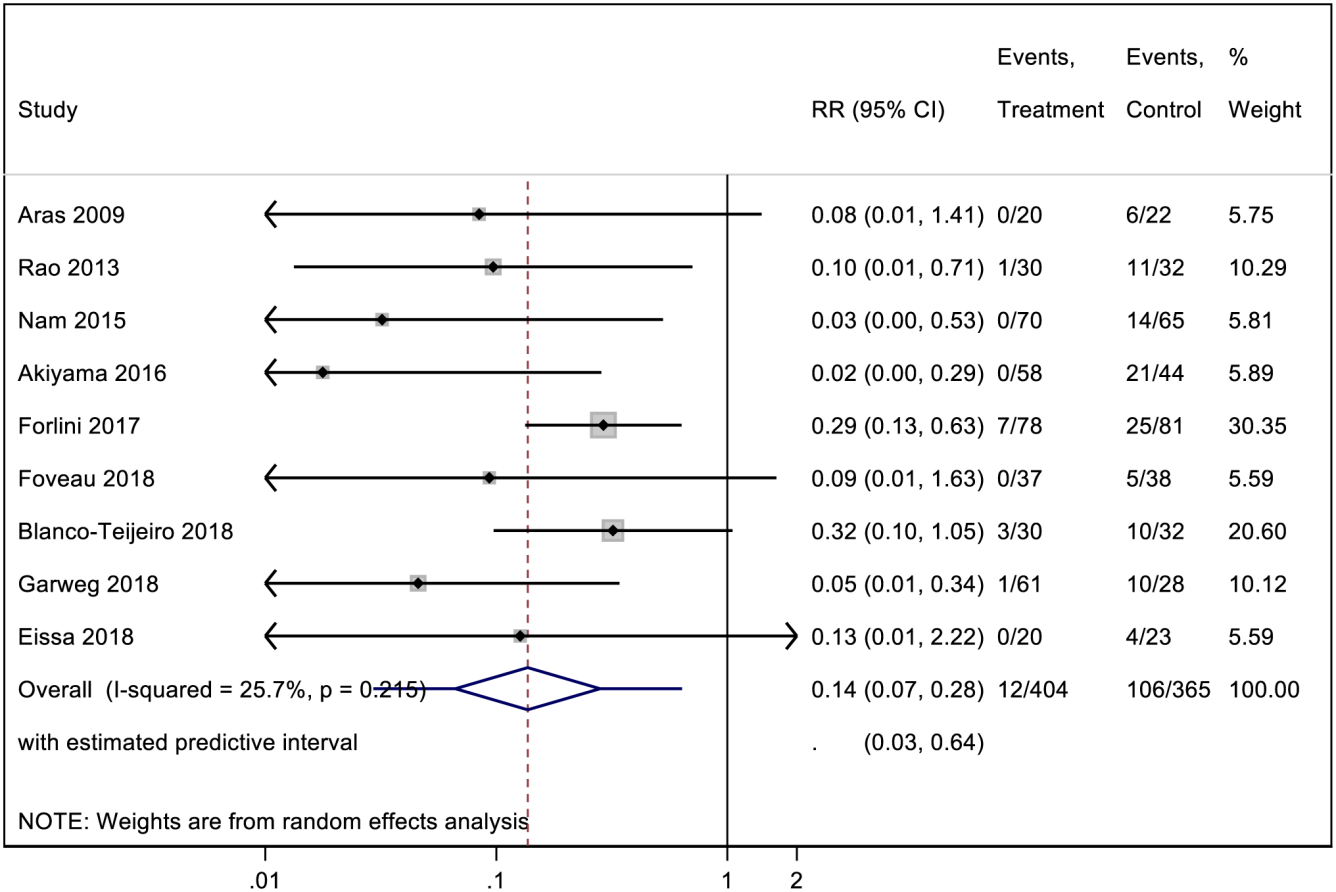
A forest plot showing the rate of macular epiretinal membrane development following ILM peeling and non-ILM peeling vitrectomy.

Pooled analysis of cohorts from all 9 studies was conducted for postoperative ERM formation. Overall, ERM developed in 12 out of 404 (3%) and 106 out of 365 (29%) eyes in ILM peeling and non-ILM peeling groups, respectively. Results from our meta-analysis are shown in Fig 2. The pooled analysis indicated a significant lower rate of postoperative ERM formation in the ILM peeling group than in the non-ILM peeling group (RR estimate = 0.14; CI: 0.07 to 0.28; P< 0.001). No statistical heterogeneity was found between the two groups (I^2^ = 25.7%; P = 0.21).

### Mean BCVA change

**Fig 3:**
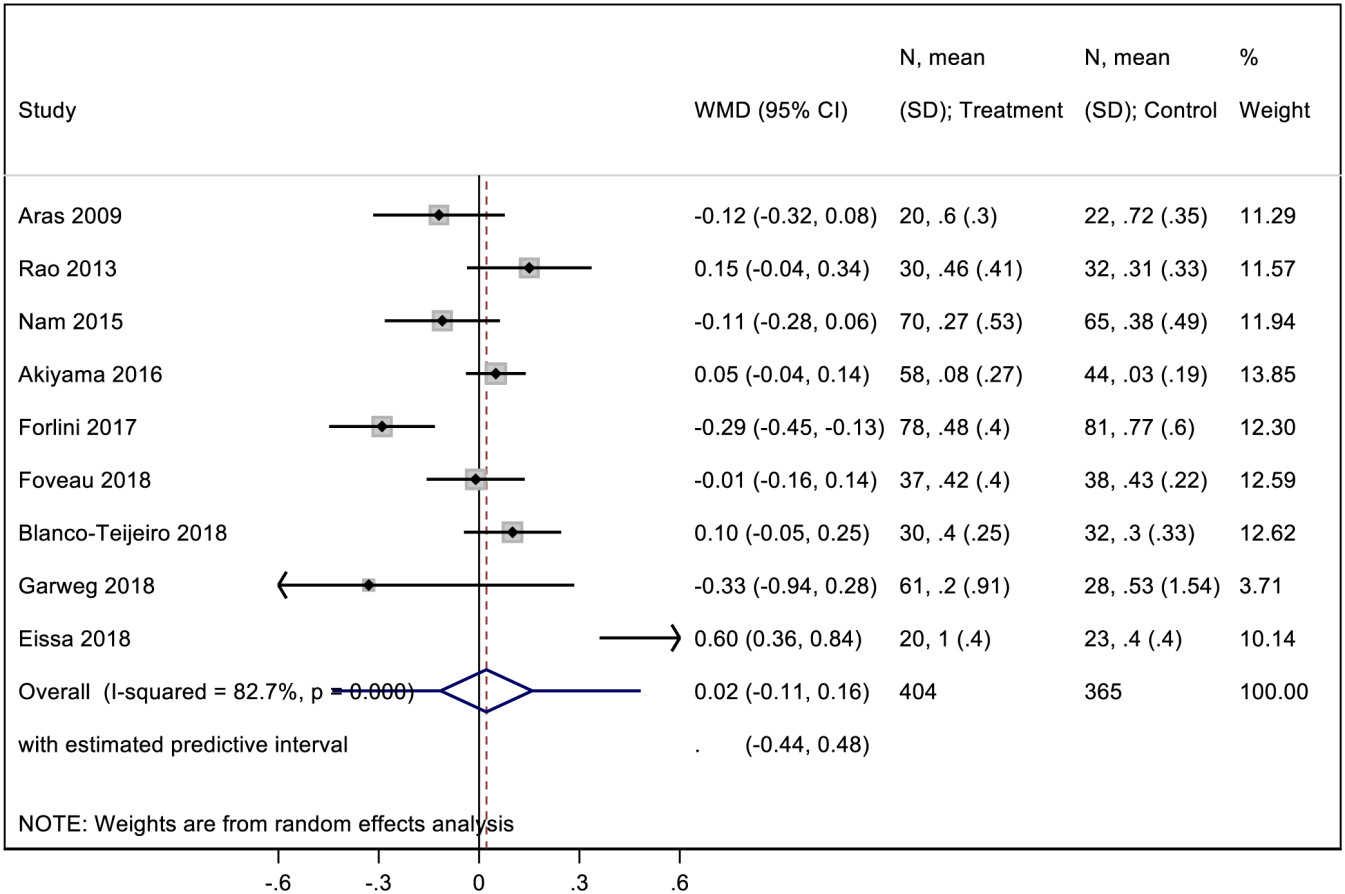
A forest plot showing mean BCVA change following ILM peeling and non-ILM peeling vitrectomy.

Pooled analysis of cohorts from all 9 studies was conducted for postoperative mean BCVA change. Results from our meta-analysis comparing mean BCVA change in the two groups are displayed in Fig 3. The pooled analysis revealed no statistical difference in mean BCVA change between the ILM peeling group and the non-ILM peeling group (WMD = 0.02; CI: -0.11 to 0.16; P = 0.75). There was significant heterogeneity between studies (I^2^ = 82.7%; P< 0.001).

### Rate of recurrence of retinal detachment

**Fig 4:**
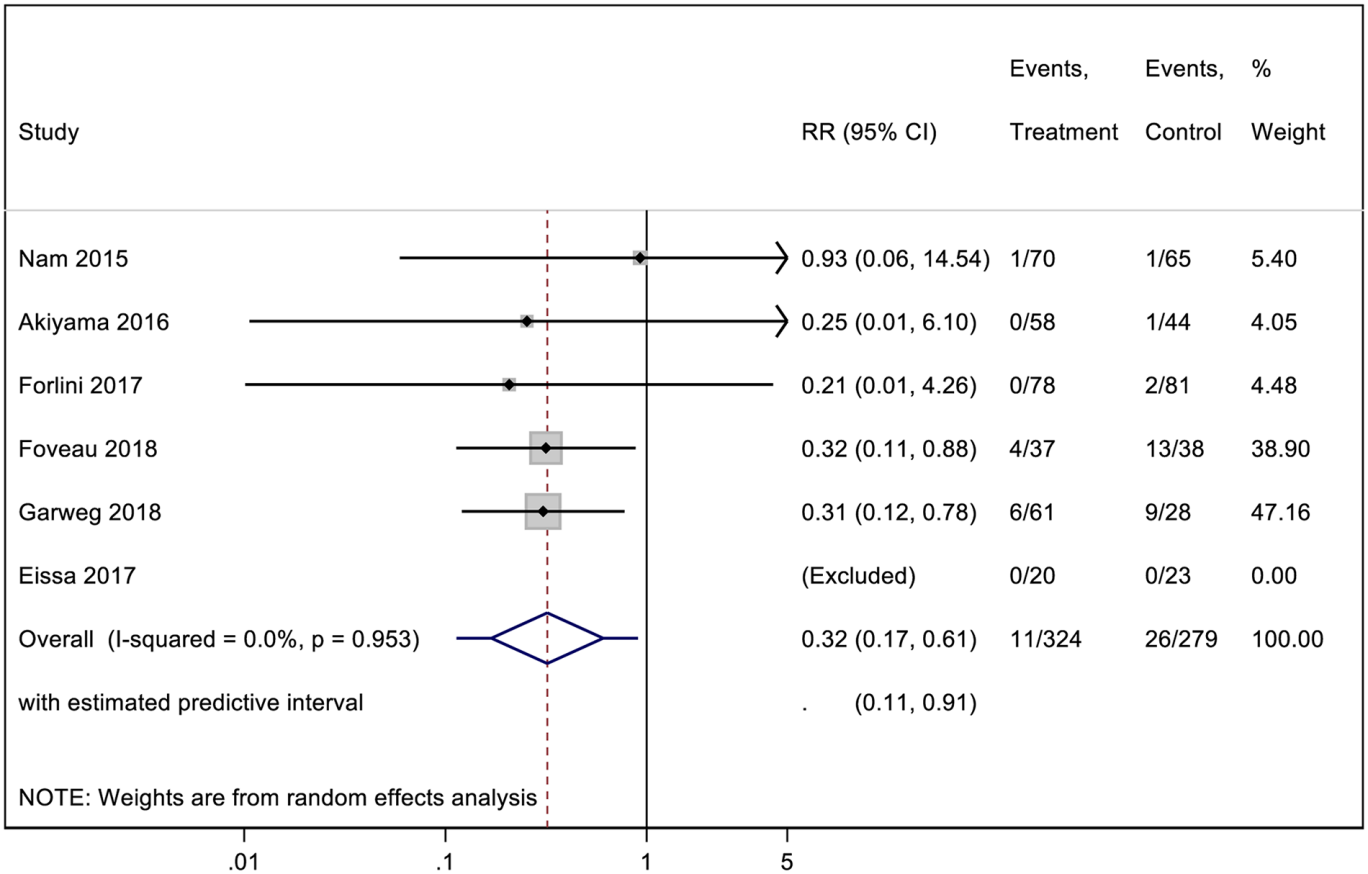
A forest plot showing the rate of recurrence of retinal detachment following ILM peeling and non-ILM peeling vitrectomy.

Pooled data for investigating the rate of RD recurrence were obtained from 6 studies. Overall, a total of 11 out of 324 (3.4%) eyes had recurrence of RD after vitrectomy in the ILM peeling group and 26 out of 279 (9.3%) in the non-ILM peeling group. Results from our meta-analysis comparing rate of RD recurrence between the group with ILM peeling and the group without ILM peeling are shown in Fig 4. The pooled analysis yielded statistical difference in the rate of RD recurrence between the two groups (RR = 0.32; CI = 0.17 to 0.61; P< 0.001), with no significant heterogeneity (I^2^ = 0%; P = 0.95).

## Discussion

The results of this systematic review and meta-analysis of published studies that compared vitrectomy with ILM peeling versus non-ILM peeling vitrectomy in patients with RRD, revealed a significantly lower incidence of postoperative macular ERM in patients who received ILM peeling at the time of vitrectomy, whereas no difference was found in visual outcome.

The purpose of this study was to identify the effects of ILM peeling versus non-ILM peeling during vitrectomy for RRD, looking at two main outcomes: the rate of ERM development and visual change.

Regarding the first outcome, the results of this meta-analysis revealed that performing ILM peeling determined a significant and consistent effect in protecting against ERM development after vitrectomy for RRD (RR: 0.1), with an overall rate of ERM development after vitrectomy of 3% in the ILM peeling group and 29% in the non-ILM peeling group. The large difference between the two groups reflects clinical evidence of ILM peeling in preventing ERM onset, being supported by several reasons: 1) high statistical difference between the two groups, with a small confidence interval; 2) even if there was some difference between studies when considering clinical and surgical variables, which have been associated with incidence of ERM, in each study no significant difference was found at baseline between the two cohorts of patients; and 3) Optical Coherence Tomography was used for the diagnosis of ERM in all studies but one, which based ERM diagnosis on a biomicroscopic fundus examination, with the result of minimizing bias of ERM diagnosis.

A possible explanation for the preventive effect of ILM peeling on ERM development may be found when looking at the pathogenesis of ERM. Internal limiting membrane has been assumed to provide retinal pigment epithelial cells and other progenitor cells with a suitable support for proliferation and formation of ERM [3–12]. Peeling of the internal limiting membrane has been demonstrated to completely remove the posterior vitreous cortex, cellular component and extracellular matrix [18,19]. Conversely, an undetached vitreous cortex has been reported in 20% of patients receiving vitrectomy without ILM peeling for RRD [20].

Even if it is sometimes asymptomatic, ERM has been considered one of the most frequent causes of metamorphopsia and visual loss after successful RRD repair [21,22]. In particular, following a vitrectomy for RRD, such a large proportion of patients with ERM complain of impaired vision that ERM removal surgery is required, ranging between 33% and 85% of cases [12,21].

However, the efficacy of ILM peeling in improving postoperative BCVA compared with non-ILM peeling after vitrectomy for RRD, is still debatable.

Internal limiting membrane peeling leads to mechanical trauma of retinal micro-structures, which could result in the following: dissociated optic nerve fiber layer, dimple sign, pitting, temporal macular thinning, concentric macular dark spots, and forceps-related retinal thinning [23]. Nonetheless, the effect of this anatomical damage on visual function is still controversial [24–27].

The results of our meta-analysis showed no significant difference in visual change after a minimum follow-up of six months between eyes receiving ILM peeling and those without ILM peeling. Among the included studies, 4 out of 9 [3,4,8,9] described a trend towards better visual acuity in the ILM peeling group, with statistical significance in one study; on the contrary, 4 studies [5–7,11] demonstrated a trend toward worse visual acuity in the ILM peeling group, with statistical significance reported only in the RCT, which highlighted a better visual acuity change in patients without ILM peeling. The remaining study [10] showed the same visual outcome between the two procedures.

These findings indicate that both groups had a comparable visual outcome, despite the fact that 29% of eyes in the non-ILM group suffered from macular ERM.

A final conclusion cannot be drawn because of difficulties in adjusting possible variables which could have had an effect on visual outcome (such as duration of surgery, status of the macula at the time of diagnosis, duration of macular detachment, and type of dye used for ILM peeling). Nevertheless, from the visual result of this meta-analysis we hypothesize a possible injury related to the maneuver of ILM peeling, especially when considering the higher incidence of macular ERM in the non-ILM peeling cohort that should have led to lower BCVA.

The effect of ILM peeling on visual function should be referred to the anatomic condition of the macula as well. In other macular pathologies, such as diabetic macular edema, ILM peeling seems to cause damage related to the modifications of Muller cells, in particular when the edema has become chronic [28]. Likewise, it is plausible to assume that the macular condition in RRD may play a relevant role for visual outcome after peeling.

Moreover, the effect of ILM peeling on visual outcome should be assessed also considering mean time of follow-up after surgery. A recent meta-analysis study, which compared clinical outcomes of ILM peeling versus non-ILM peeling during vitrectomy for idiopathic ERM, demonstrated significantly better BCVA in the non-ILM peeling group at the 12 month follow-up, whereas this result was inverted after an 18 month follow-up with BCVA significantly better in the ILM peeling group [29].

Four studies [6,8–10] in our analysis had a mean follow-up of 6 months or longer, while 5 studies [3–5,7,11] had a follow-up of at least one year. This period represents a fair follow-up time for evaluating the effect of the two different surgical procedures on visual function, since roughly 5 months have been reported as the mean post-vitrectomy timeframe for ERM to cause symptoms that require further surgery for removal [12]. However, it cannot be excluded that a longer follow-up time could yield different results.

When evaluating the recurrence rate of RD, a statistical difference has been demonstrated by this meta-analysis, with a lower rate of RD recurrence in the ILM peeling group. Although data on RD recurrence were reported from six studies [3–6,8,10], five studies were included in the pooled analysis since Eissa et al [6] were excluded as there was no case of RD recurrence in both groups. Rao et al [7] did not examine the rate of RD recurrence in their study, while Blanco-Teijeiro et al [11] and Aras et al [9] defined recurrence of retinal detachment as an exclusion criteria from their retrospective analysis. Although the present meta-analysis shows that ILM peeling reduces the rate of RD recurrence, this finding has to be considered cautiously since it was markedly influenced by two studies [8,10], which reported the highest rates of RD recurrence in both groups.

The present study has some limitations. First, only one RCT was included whilst most of the enrolled studies were retrospective. Hence, bias may have been introduced. Second, individual patient data were not available and the analysis was conducted on the tabulated data. Third, included studies could present some variability in surgical and clinical characteristics. However, the study of meta-analysis presents higher power and more accurate confidence intervals than those from an individual report. Moreover, the following characteristics featured in our meta-analysis: all included studies showed a low-to-moderate risk of bias according to the Newcastle-Ottawa Quality Assessment Scale; funnel plots, Harbord’s and Egger’s test demonstrated no evidence of publication bias; and there was no difference in clinical characteristics between the cohorts of each study at baseline.

In conclusion, our meta-analysis showed that ILM peeling during vitrectomy for RRD is efficient in preventing the formation of macular ERM postoperatively, with a lower rate of recurrence of RD compared to non-ILM peeling vitrectomy. Despite such a positive anatomical result, no corresponding visual benefit was found, as visual change was comparable between the two groups. Therefore, further large RCTs are needed to yield valid results and corroborate our conclusions, as well as identifying which eyes will be at high risk of developing an ERM after vitrectomy for RD in order to perform the peeling in this category of patients.

## Supporting information

S1 Table PRISMA checklist 2009. (DOCX)

S2 Table Search strategy. (DOCX)

S3 Table Quality assessment by using Newcastle-Ottawa scale. (DOCX)

S1 Fig Funnel plot for the rate of ERM development. (TIF)

S2 Fig Funnel plot for the rate of RD recurrence. (TIF)

S3 Fig Funnel plot for mean BCVA change. (TIF)
