# Supplementary material for: Internal limiting membrane peeling versus no peeling during primary vitrectomy for rhegmatogenous retinal detachment: A systematic review and meta-analysis
Source: PLoS One. 2018 Jul 19;13(7):e0201010. doi: 10.1371/journal.pone.0201010 (PMC6053210; doi:10.1371/journal.pone.0201010)
Supplement: S2 Table — (DOCX) [file pone.0201010.s002.docx]

**S2 Table. Search strategy.**

| **Relevant text of ILM peeling**   1. Internal limiting membrane 2. Inner limiting membrane 3. ILM 4. Peeling 5. 1 and 4 or 2 and 4 or 3 and 4 6. Vitrectomy with internal limiting membrane peeling 7. Vitrectomy with inner limiting membrane peeling 8. 5 or 6 or 7 | **Relevant text of Retinal Detachment**   1. Retinal detachment     **Combined (Final strategy)**   1. 8 and 9 |
| --- | --- |

Web sites and uniform resource locator:

**PubMed**: <http://www.ncbi.nlm.nih.gov/pubmed>

**Embase**: https://www.embase.com

**Cochrane:** http://www.cochranelibrary.com

**Web of science**: http://www.webofknowledge.com

**ClinicalTrials.gov.**: <https://clinicaltrials.gov>

**Medline**: <http://www.ovid.com/site/catalog/databases/901.jsp>
