# Supplementary material for: Internal limiting membrane peeling versus no peeling during primary vitrectomy for rhegmatogenous retinal detachment: A systematic review and meta-analysis
Source: PLoS One. 2018 Jul 19;13(7):e0201010. doi: 10.1371/journal.pone.0201010 (PMC6053210; doi:10.1371/journal.pone.0201010)
Supplement: S3 Table — (DOCX) [file pone.0201010.s003.docx]

S3 Table: Newcastle-Ottawa Scale score for non-randomized studies

|  | Selection  ( max 4 stars) | Comparability  ( max 2 stars) | Exposure  ( max 3 stars) | Total score |
| --- | --- | --- | --- | --- |
| Aras et al 2008 | 3 | 1 | 2 | 6 |
| Rao et al 2013 | 3 | 1 | 2 | 6 |
| Nam et al 2015 | 3 | 1 | 3 | 7 |
| Akiyama et al 2016 | 3 | 1 | 3 | 7 |
| Forlini et al 2017 | 3 | 1 | 3 | 7 |
| Garweg et al 2018 | 3 | 1 | 3 | 7 |
| Blanco-Teijeiro et al 2018 | 3 | 1 | 3 | 7 |
| Foveau et al 2018 | 3 | 1 | 3 | 7 |
