# Supplementary figures and images for: Internal limiting membrane peeling versus no peeling during primary vitrectomy for rhegmatogenous retinal detachment: A systematic review and meta-analysis
Source: PLoS One. 2018 Jul 19;13(7):e0201010. doi: 10.1371/journal.pone.0201010 (PMC6053210; doi:10.1371/journal.pone.0201010)

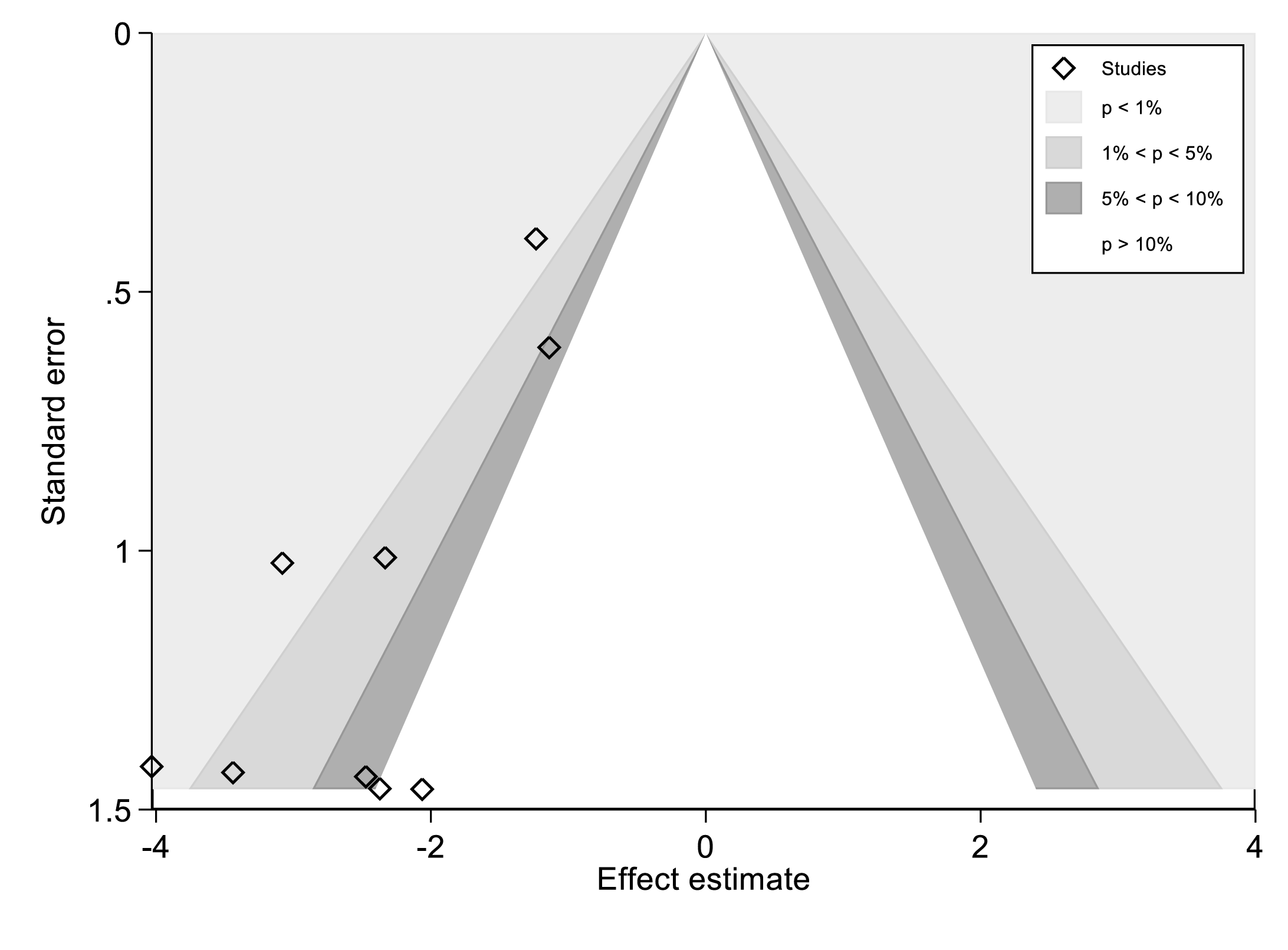

Supplement: S1 Fig — (TIF) [file pone.0201010.s004.tif]

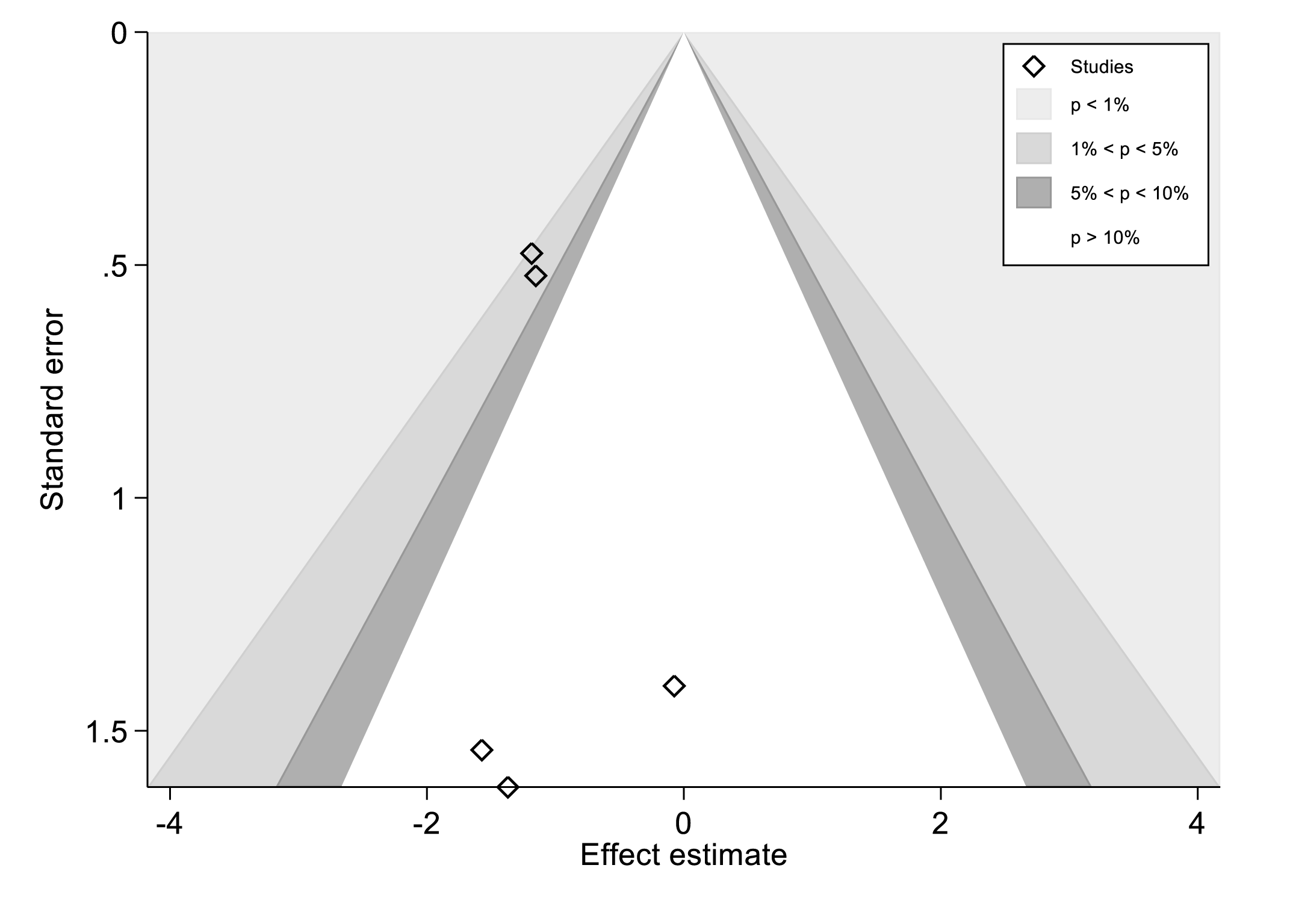

Supplement: S2 Fig — (TIF) [file pone.0201010.s005.tif]

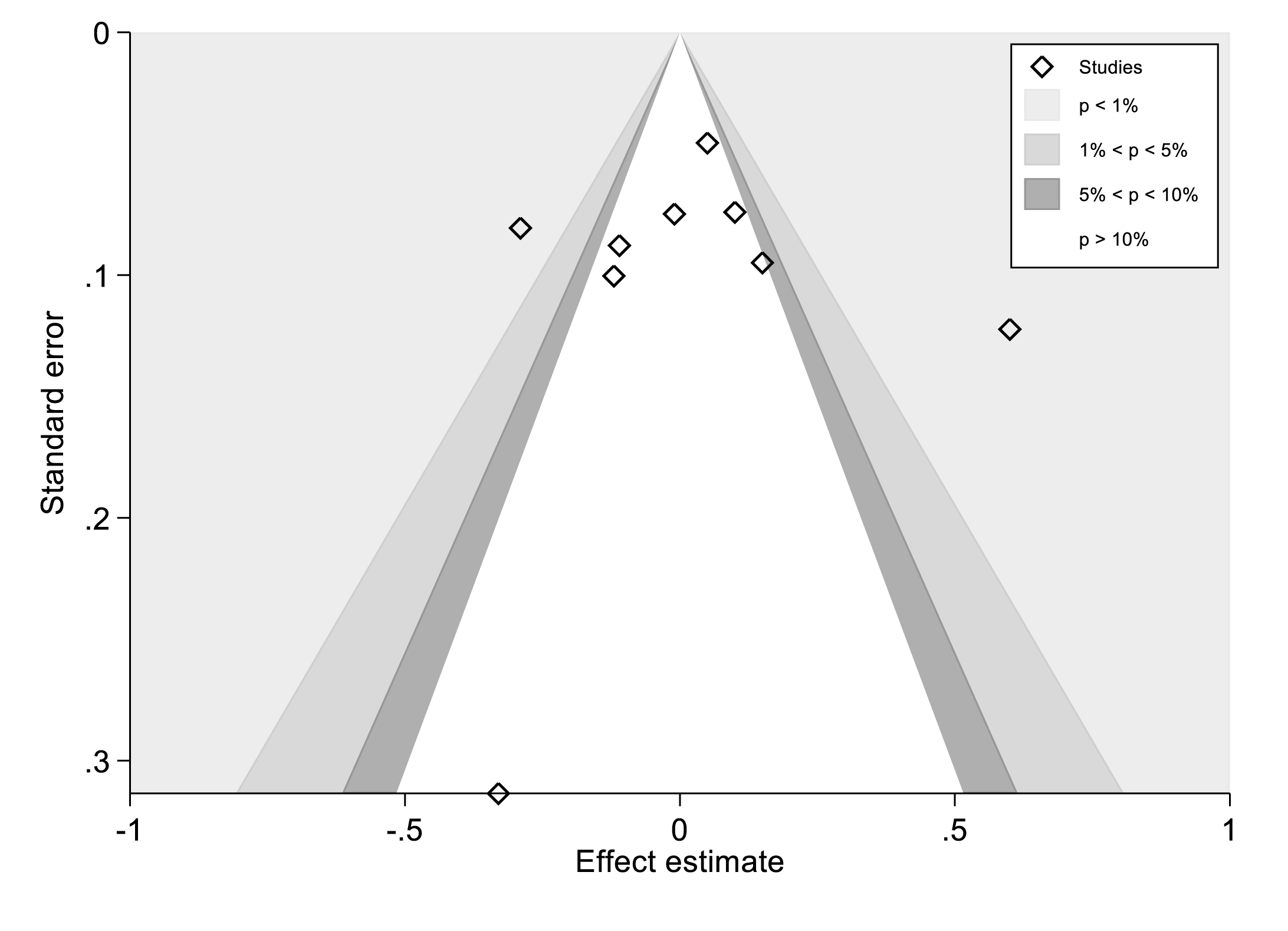

Supplement: S3 Fig — (TIF) [file pone.0201010.s006.tif]
